# Morphological Variability and Distinct Protein Profiles of Cultured and Endosymbiotic *Symbiodinium* cells Isolated from *Exaiptasia pulchella*

**DOI:** 10.1038/srep15353

**Published:** 2015-10-20

**Authors:** Buntora Pasaribu, Li-Chi Weng, I-Ping Lin, Eddie Camargo, Jason T. C. Tzen, Ching-Hsiu Tsai, Shin-Lon Ho, Mong-Rong Lin, Li-Hsueh Wang, Chii-Shiarng Chen, Pei-Luen Jiang

**Affiliations:** 1 Graduate Institute of Marine Biology, National Dong-Hwa University, Pingtung, 944 Taiwan; 2 Taiwan Coral Research Center, National Museum of Marine Biology and Aquarium, Pingtung 944, Taiwan; 3 Graduate Institute of Biotechnology, National Chung-Hsing University, Taichung 402, Taiwan; 4 Department of Marine Biotechnology and Resources, National Sun Yat-Sen University, Kaohsiung 804, Taiwan; 5 Department of Agronomy, National Chia-Yi University, Chia-Yi 600, Taiwan; 6 Department of Biotechnology, National Formosa University, Taiwan

## Abstract

*Symbiodinium* is a dinoflagellate that plays an important role in the physiology of the symbiotic relationships of Cnidarians such as corals and sea anemones. However, it is very difficult to cultivate free-living dinoflagellates after being isolated from the host, as they are very sensitive to environmental changes. How these symbiont cells are supported by the host tissue is still unclear. This study investigated the characteristics of *Symbiodinium* cells, particularly with respect to the morphological variability and distinct protein profiles of both cultured and endosymbiotic *Symbiodinium* which were freshly isolated from *Exaiptasia pulchella*. The response of the cellular morphology of freshly isolated *Symbiodinium* cells kept under a 12 h L:12 h D cycle to different temperatures was measured. Cellular proliferation was investigated by measuring the growth pattern of *Symbiodinium* cells, the results of which indicated that the growth was significantly reduced in response to the extreme temperatures. Proteomic analysis of freshly isolated *Symbiodinium* cells revealed twelve novel proteins that putatively included transcription translation factors, photosystem proteins, and proteins associated with energy and lipid metabolism, as well as defense response. The results of this study will bring more understandings to the mechanisms governing the endosymbiotic relationship between the cnidarians and dinoflagellates.

Symbiotic associations are mostly found in marine environments. A well-known example of which is the mutualistic symbiosis of cnidarians (coral and sea anemone) with the dinoflagellate, *Symbiodinium* sp^1^. The mutualistic association involves the translocation of 90% of the photosynthetically fixed carbon in *Symbiodinium* into the host cytoplasm, and in return, the host provides nutrients for the dinoflagellates to live and grow^2^. It has been proposed that this intracellular symbiosis (i.e. endosymbiosis) plays key role in the maintenance of coral metabolism and health.

*Symbodinium* has been found as a symbiont in cnidaria and other invertebrates^3^,^4^,^5^. Endosymbiotic *Symbiodinium* is widely distributed throughout the host cnidarian’s gastrodermis cell layer, at a density of 10^6^ per cm2 of colony surface area^6^,^7^. Recently, *Symbiodinium* was divided into nine major lineages (clade A-I) based on the analysis of the 18 S-rDNA and internal transcribed spacer regions (ITS)^8^,^9^. Symbiodinium genetic variability may reflect the different features of *Symbiodinium* functional biology and responses to environmental stress^10^. For instance, clade D is able to resilient to coral bleaching event due to the tolerance to thermal stress^11^. Using a proteomic approach, it has been shown that the different protein expression of the cultured and the symbiotic *Symbiodinium* cells were observed in sea anemone^12^. After examining the culture of symbiotic *Symbiodinium* from host, Krueger and colleague have successfully cultivated endosymbiont C15 by mimicking the host environment *ex hospite*^13^. However, the cellular and molecular mechanisms governing this mutualism are not yet fully understood.

Environmental stress, such as temperature, salinity, light intensity and disease, are causing disruption of the coral-dinoflagellate symbiotic association^14^,^15^,^16^. Temperature is one of the major causes affecting the stability of endosymbiotic associations^17^,^18^. Nevertheless, some previous works have shown that both low and high temperatures could induce the physiological impact on coral such as bleaching and mortality^19^. It has been reported that the exposure to temperature at 30–31 °C in the seawater, southern Taiwan, caused the coral bleaching^20^,^21^. Moreover, when the coral was exposed to 14 °C with full sunlight, the photosynthetic ability of the *Symbiodinium* was reduced, and coral become bleached^22^. Nonetheless, the morphological profile, adaptation behavior and survival rate of the free-living *Symbiodinium* against thermal stress remains to be clarified.

In order to elucidate the mechanism allowing endosymbiotic *Symbiodinium* in clade B to adapt to *ex hospite* conditions and extreme temperatures, endosymbiotic *Symbiodinium* was isolated from host gastrodermal cells and purified with different percentages of Percoll gradient solution. Then, the *Symbiodinium* was inoculated onto the medium to obtain the pure *Symbiodinium* culture, and the clade identification was carried out. *Symbiodinium* cell proliferation progressed more slowly when they were subjected to extreme temperatures (30 °C; 15 °C) than when they were cultured at normal temperatures (25 °C). However, morphological observations revealed that when symbiont *Symbiodinium* were cultivated *ex hospite*, their size changed. The twelve distinct protein profiles derived from the endosymbiotic *Symbiodinium* cells putatively included transcription translation factors, photosystem proteins, and proteins associated with energy and lipid metabolism as well as defense response.

## Results and Discussion

### Morphological alterations of Exaiptasia pulchella in response to stress-induced bleaching

**Figure 1:**
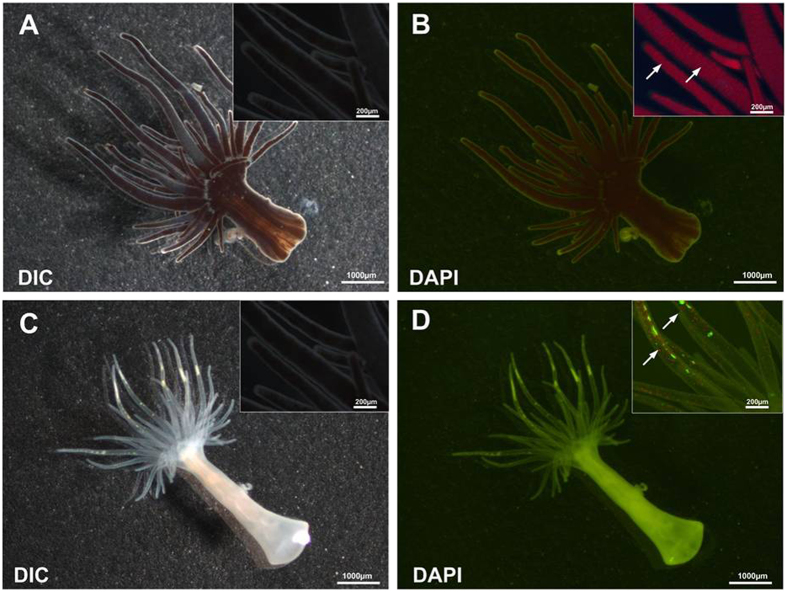
Microscopic examination of healthy and bleached in *Exaiptasia pulchella*. (**A**) Healthy *Exaiptasia pulchella* were observed under light microscopy to be brown in color throughout the column and tentacles. (**B**) Fluorescence microscopy under a DAPI filter shows the red auto-fluorescence representing the cellular chlorophyll of *Symbiodinium* sp. cells (white filled arrowheads). (**C**) Bleached *Exaiptasia pulchella* were observed using light microscopy. (**D**) Bleached *Exaiptasia pulchella* tissue observed via fluorescence microscopy revealed only a small amount of red auto-fluorescence, at the tentacle edges (white filled arrowheads).

**Figure 2:**
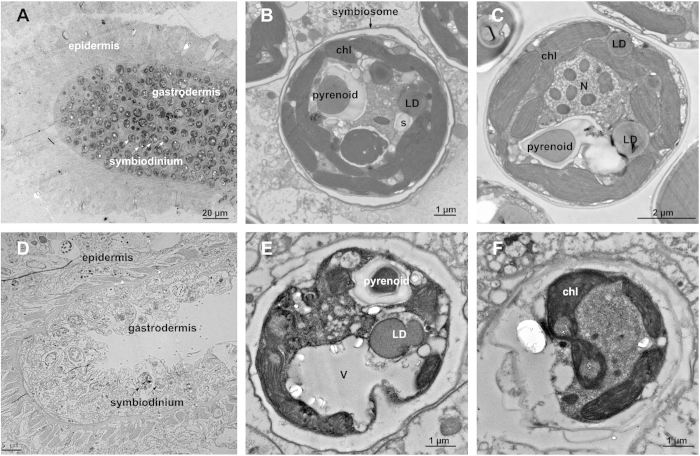
Morphological profiles of an *Exaiptasia pulchella* tentacle and *Symbiodinium* cells, observed via Transmission Electron Microscopy. (**A,D**) Ultrastructure of *E. pulchella* tentacle, healthy vs bleached *Symbiodinium* cells residing inside the host, observed via transmission electron microscopy (**B,C**). *Symbiodinium* ultrastructure in a bleached tentacle (**E,F**). Abbreviations: LD, lipid droplet; Ch, chloroplast; Pyr, pyrenoids; N, nucleolus; acc: accumulation body.

Endosymbiotic stability is dependent on the regulation of *Symbiodinium* cells living in the host gastrodermal cell layer^15^,^23^. *Exaiptasia pulchella*^24^ is a sea anemone that is widely distributed in the tropical and subtropical Pacific Ocean. It can be easily cultured in the laboratory and commonly used as a model organism for studying the cnidarian-*Symbiodinium* mutualism^25^,^26^,^27^. *Symbiodinium* cells appear brown in color, and thus they bring about the apparent color of *E. pulchella* (Fig. 1A). In contrast, we have shown that the environmental stress caused the bleaching of sea anemone tentacles due to the loss of *Symbiodinium* cells, leading to the bleaching of the coral (Fig. 1C). It has been reported that unfavorable environmental conditions such as elevated temperature, extreme pH, nutrient deprivation, and changes in salinity could create conditions inducing the collapse of the symbiotic relationship, resulting in the loss of *Symbiodinium*^14^,^28^,^29^,^30^,^31^,^32^. Bleached anemones lost 86% of their *Symbiodinium* symbionts^32^, a phenomenon that was visualized by fluorescence microscopy to detect the auto-fluorescence of *Symbiodinium* chlorophyll, in order to show that *Symbiodinium* cell density decreased in response to environmental stress (Fig. 1B,D). During bleaching, the amount of green fluorescence in the host body and tentacles increased significantly (Fig. 1D). The difference between healthy and unhealthy *E. pulchella* can be visualized clearly under electron microscope (Fig. 2A,D). Ultrastructural observations of healthy sea anemone tentacles revealed the presence of abundant *Symbiodinium* cells in the gastrodermal layer, as the *Symbiodinium* intracellular structures, such as chloroplasts, nuclei, pyrenoids and lipid droplets, were noted (Fig. 2). Symbiotic cells are usually in coccoid forms surrounded by a cellulose-based cell wall. They inhabit a specialized host vacuole called a symbiosome (Fig. 2B). The signs of structural degradation of the *Symbiodinium* cells in the bleached *E. pulchella* were shown. This study also revealed the occurrence of the reduction in gastrodermal *Symbiodinium* densities (Fig. 2A,D). Presumably, the *Symbiodinium* cells were degraded during the bleaching period (Fig. 2D). Interestingly, the major characteristics of these symbiotic cells included the following: the presence of only a few organelles inside of a large vacuole containing numerous lipid droplets, the presence of cytoplasmic debris in the lumen, and loosened thylakoids (Fig. 2E,F). These morphological observations were similar to the previously reported observations of the morphological changes in both the host and *Symbiodinium* during bleaching^33^,^34^.

### Isolation of Symbiodinium cells from Exaiptasia pulchella and their culture in medium

**Figure 3:**
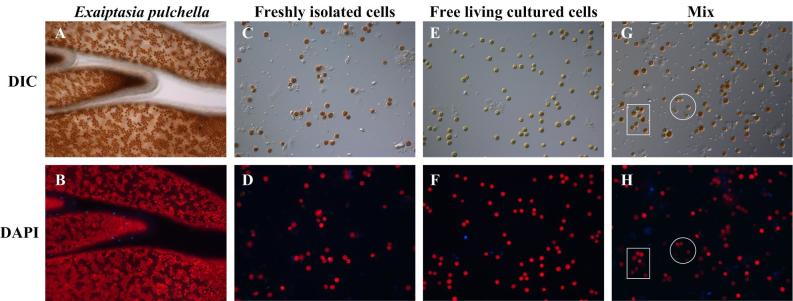
Freshly isolated *Symbiodinium* cells and free-living cultured cells were observed using light microscopy. (**A**) Brown *Symbiodinium* residing in the *Exaiptasia pulchella* tentacle. (**B**) Fluorescence microscopy of *Exaiptasia pulchella*. (**C**,**E**,**G**) light microscopy of freshly isolated S*ymbiodinium*, free-living cultured *Symbiodinium* sp., mixed cells of freshly isolated (rectangular white box), and free-living S*ymbiodinium* (circular white box), respectively. (**D**,**F**,**H**) Freshly isolated S*ymbiodinium*, free-living cultured *Symbiodinium*, mixed cells of freshly isolated (rectangular white box), and free-living S*ymbiodinium* (circular white box), respectively, were observed using fluorescence microscopy. Little red auto-fluorescence was detected, representing the cellular S*ymbiodinium* chlorophyll.

**Figure 4:**
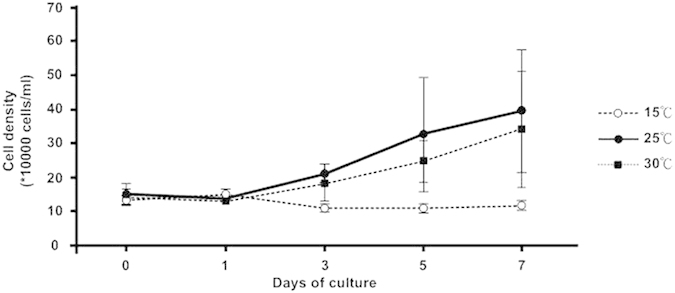
Growth curves of *Symbiodinium* cells cultivated at different temperatures. Temperature treatments (15 °C, 25 °C and 30 °C) were applied to free-living *Symbiodinium* sp. The data represents means ± SD (n = 3).

Many symbiotic dinoflagellates can be isolated and maintained in culture, given the presence of suitable conditions. Most investigators have concluded that the morphology and life cycle of the free-living and symbiotic forms are fundamentally different to each other, but few details are known. The establishment of free-living *Symbiodinium* populations could help to preserve both *Symbiodinium* and coral during the bleaching periods. Freshly isolated *Symbiodinium* cells were collected from *E. pulchella* via a Percoll gradient^35^ (Fig. 3C,D). All remaining host tissue debris was removed from the *Symbiodinium* cells, after which the cells were incubated in f/2 medium in filtered seawater. The *Symbiodinium* isolates were successfully maintained by culturing them in f/2 medium (Fig. 4). After being cultured for a few months, some of the *Symbiodinium* cells died (appeared as blue fluorescence); however, most *Symbiodinium* cells in the culture medium were still alive (appeared as red fluorescence) (Fig. 3E,F). Studies have shown that the *Symbiodinium* clades or types associated with corals are different, depending on environmental conditions^36^. The *Symbiodinium* clade identification was conducted via RFLP, using restriction enzymes, *Taq*1, and *Sau* 3A1. The results showed that clade B was dominant in both freshly isolated and free-living cultured *Symbiodinium* cells. In the present study, the physiology and molecular response of symbiotic *Symbiodinium* to environmental stress was investigated by isolating fresh symbiotic cells from the host sea anemone. Here, the comparison of the freshly isolated with the free-living cultured cells revealed the differences in the color and size (Figs 2 and 3). Freshly isolated *Symbiodinium* cells had a diameter of 6.49 ± 1.06 μm, which became smaller after culturing in the medium, with a diameter of 5.6 ± 0.02 μm (Fig. 3G, Table 1). The chloroplast content in the *Symbiodinium* cells decreased, resulting in a more light brown color than the freshly isolated cells (Figs 2C,G and 3C). Similarly, free-living cultured *Symbiodinium* cells isolated from sea anemones were shown to be compact in structure and morphologically different from the *Symbiodinium* cells residing in the host (Fig. 2). The morphological differences of free-living cultured *Symbiodinium* have been reported previously^37^.

### Extreme temperature stress induced changes in the cell morphology of cultured Symbiodinium cells

**Figure 5:**
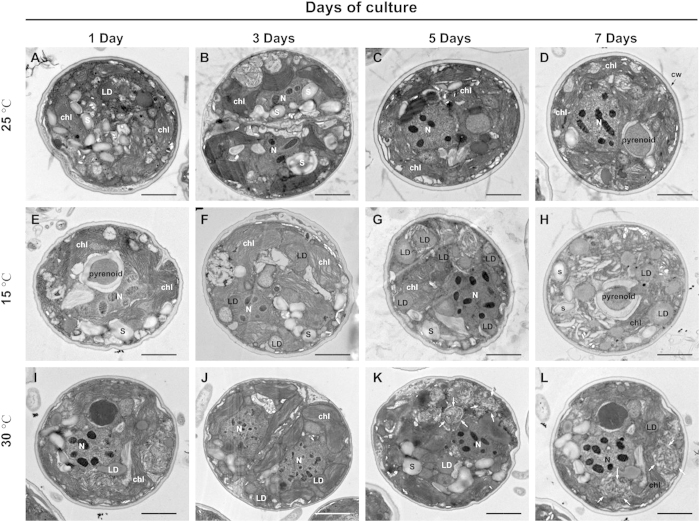
TEM micrographs showing ultrastructural changes during the 7-day treatment at different temperatures in *Symbiodinium* cells. *Symbiodinium* cells were cultured at a normal temperature (25 °C) for the indicated times (**A–D**). *Symbiodinium* cells were cultured at a low temperature (15 °C) for the indicated times (**E–H**). *Symbiodinium* cells were cultured at a high temperature (30 °C) for the indicated times (**I–L**). Abbreviations: LD, lipid droplet; Ch, chloroplast; Pyr, pyrenoids; N, nucleolus. The scale bars indicate 2 μm.

In the free-living cultures, *Symbiodinium* could freely approach the nutrients in the medium. Although it was likely that the nutrient uptake mechanism changed under such culturing condition, it was determined that it would not be responsible for the physiological changes observed in *Symbiodinium* cells^37^. To examine the effect of extreme temperature on *Symbiodinium* cellular growth, free-living *Symbiodinium* cells were cultivated at low or high temperatures for 7 days. The cells cultivated at low temperatures (15 °C) proliferated more slowly than those cultivated at normal temperatures (Fig. 4). Culturing at 25 °C and 30 °C did not cause any influence on the growth of *Symbiodinium* cells (Fig. 4), but numerous lipid droplets started to be accumulated, and then they were disrupted after the 5^th^ day of high-temperature treatment. It was similarly reported that the response to changes in temperature was an adaptive physiological adjustment of *Symbiodinium*^28^,^38^. Furthermore, at a lower temperature (15 °C), the *Symbiodinium* cells stopped proliferating (Fig. 4), and lipid droplets accumulated and occupied most of the cytoplasm in the *Symbiodinium* cells from day 3 (Fig. 5E–H). The *Symbiodinium* cells started proliferating at day 3 and were structurally degraded after 5 days at 30 °C. At this stage, the cells contained highly disrupted lipid droplets (Fig. 5K,L, arrows). Fewer lipid droplets were present in the *Symbiodinium* cells cultured at normal temperatures (25 °C) (Fig. 2A–D). The *Symbiodinium* cells were structurally degraded after 7 days at 30 °C and contained highly disrupted lipid droplets (Fig. 2I, arrows). Fewer lipid droplets were present in cells cultured at normal temperatures (Fig. 2G).

### Identification of specifically expressed proteins from freshly isolated symbiotic Symbiodinium cells

**Figure 6:**
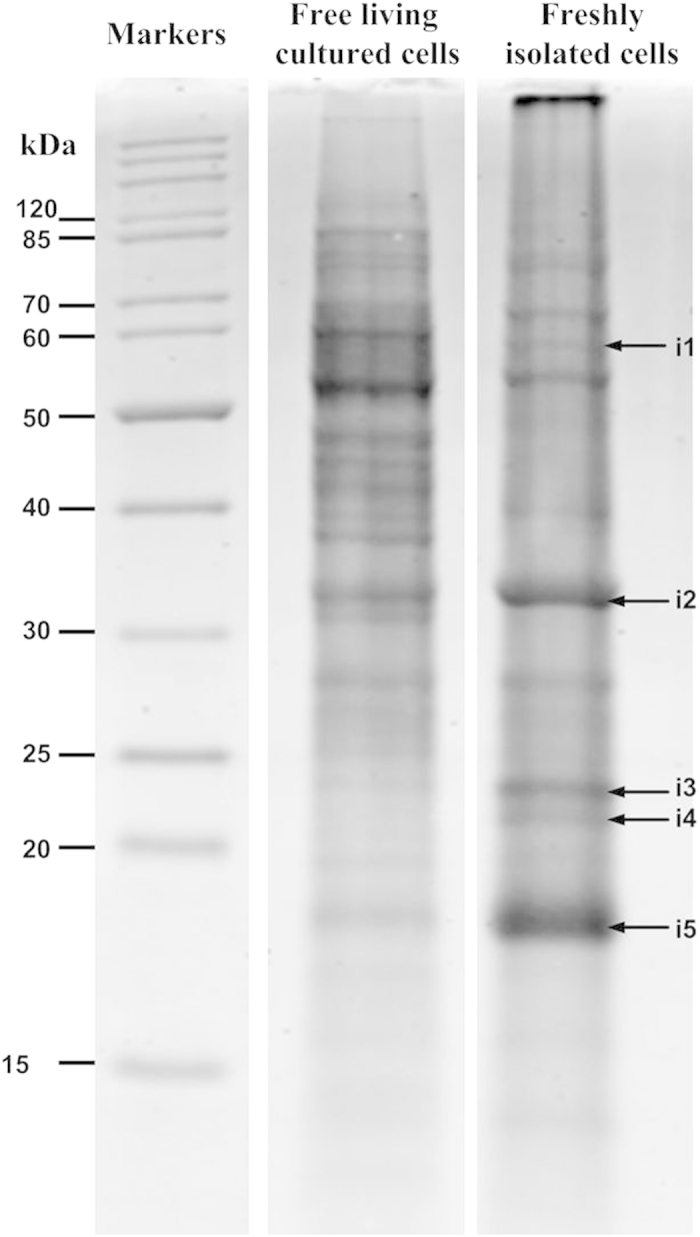
Different proteins expressed in free-living cultured and freshly isolated symbiotic *Symbiodinium* cells. Total protein (40 μg) was extracted from both freshly isolated and free-living cultured *Symbiodinium* sp. resolved in SDS-PAGE. Five protein bands were excised for mass spectrometric analysis.

Stochaj and Grossman^12^ reported that the protein profiles of free-living and endosymbiotic *Symbiodinium* cells were different to each other because the latter was obtained from sea anemone *Exaiptasia pallida*^12^. A proteomic approach was employed in order to obtain the protein profiles of freshly isolated *Symbiodinium* cells (Table 2). The protein expression of *Symbiodinium* cells was analyzed, and various differentially regulated proteins were detected. The SDS-PAGE of free-living cultured *Symbiodinium* cell proteins was compared to that of the freshly isolated cells (Fig. 6; Fig. S1). Twelve proteins with a variety of cellular functions were identified, including transcription and translation factors (“EF-1 alpha-like protein” from protein band I1). EF-1α (also known as EF1A) plays an essential role in translation, binding aminoacyl-tRNAs (aa-tRNAs) and bringing them to the A site of the ribosome. EF-1α is also known to be involved in several other cellular processes, including the ubiquitin-dependent proteolytic system^39^. A number of proteins that are stress-induced at the translation level have been identified in endosymbionts. “WRKY transcription factor” from protein band I2 was found to play an important role in regulating nutrient deficiency tolerance^40^. Previous studies have shown that these endosymbionts could survive in nutrient-limited environments, such as those with limited nitrogen levels^37^. In endosymbionts, nutrient limitation may induce the expression of the WRKY transcription factor for the tolerance control. “Dehydration responsive element binding protein (DREB)” from protein band I4. DREB transcription factors induce a set of abiotic stress-response genes responsible for maintaining the organism’s water balance and imparting tolerance to abiotic stress^41^. The “pre-mRNA splicing factor ATP-dependent RNA helicase-like protein” was determined as protein band I5. Furthermore, we detected that photosystem II D2 protein (PsbD2) and glutamate 1-semialdehyde (GSA) aminotransferase were overexpressed in endosymbiotic cells (“Photosystem II D2 protein” from protein band I1 and “glutamate-1-semialdehyde aminotransferase” from protein band I2). GSA aminotransferase catalyzes the last step of the conversion of glutamate to δ-aminolevulinate, and eight molecules of which are needed in order to synthesize a single chlorophyll molecule^42^. Other proteins associated with energy metabolism (“NADH dehydrogenase subunit F” from protein band I2, “Oxidoreductase” from protein band I2, and “ARF-related small GTPase” from protein band I5) and lipid metabolism (“acyl-coA thioesterase 13” from protein band I3 and “7-dehydrocholesterol reductase-like” from protein band I4), as well as a defense response protein (“LRR-kinase protein” from protein band I5), were also detected (Table 2). Furthermore, the result showed that the discovery of the presence of ARF-related small GTPase in endosymbiotic *Symbiodinium* is similar to the study of the lipid droplets in the nitrogen-deprived free-living *Symbiodinium* cells^37^, demonstrating that the changes in the energy metabolism of the symbiotic association with Cnidaria such as free-living *Symbiodinium* cultured in nitrogen-deficient environments.

## Conclusion

This study revealed the morphological variability and distinct protein profiles of the free-living cultured and endosymbiotic *Symbiodinium* cells isolated from *Exaiptasia pulchella*. Temperature-induced morphological changes and the variable cell proliferation were shown in free living cultured *Symbiodinium* cells. Proteomic analysis of major proteins in freshly isolated cells were found to be involved in regulation of transcription and translation, photosystem and metabolism of energy and lipid.

## Materials and Methods

### Collection of sea anemone and isolation of *Symbiodinium* cells from *E. pulchella* tissues

The sea anemone *Exaiptasia pulchella* was collected from National Museum of Marine Biology Aquarium. They were maintained in filtered seawater (FSW) at room temperature under a photosynthetically active radiation (PAR) of 40 μmol m^−2^s^−1^ in a 12-h light/12-h dark (12L/12D) cycle. *Symbiodinium* cells isolated from its host tissue were subjected to further isolation method developed by Pasaribu *et al.*^35^. *E. pulchella* was then blended for releasing the *Symbiodinium* cells in FSW (filtered seawater) and homogenized with a glass grinder. The homogenized solution was then filtered through the 20-μm mesh and centrifuged at 4,000 × g for 5 min. The pellet collected after the centrifugation contained mostly *Symbiodinium* cells, which were resuspended in FSW and vortexed for 2 min. The mixture was centrifuged at 2,000 × g for 5 min. The pellet was retained and then resuspended in fresh FSW containing 1% (v/v) of triton X-100. The mixture was vortexed again for 2 min.

### *Symbiodinium* clade identification

The genetic identity (18S rDNA) of the cultured *Symbiodinium* was examined by PCR-RFLP (Polymerase chain reaction-Restriction fragment length polymorphism) analysis^41^, and shown to be clade B. *Symbiodinium* DNA was extracted using a plant genomic DNA extraction miniprep system (VIOGENE, Taipei). Basically, *Symbiodinium* nuclear small subunit (n18S-rDNA) was amplified by PCR from 3 replicate extracts of each of the two cultures using the primers, ss5z (an equimolar mixture of the oligonucleotides 5′-GCAGTTATAATTTATTTGATGGTCACTGCTAC-3′ and 5′-GCAGTTATAGTTTATTTGATGGTTGCTGCTAC-3′) and ss3z (5′-AGCACTGCGTCAGTCCGAATAATTCAC CGG-3′) and digested by the restriction enzymes, *Taq* I and *Sau*3A I (Promega, USA). Digestion products were separated by electrophoresis on 1.5% 0.5x TAE (Amresco, USA) agarose gels, to generate the RFLP pattern. RFLP pattern analysis was compared to the literature^41^ to assign each culture to one of the established *Symbiodinium* n18S-rDNA RFLP clades.

### *Symbiodinium* culture and treatment

The free-living *Symbiodinium* sp. (clade B) were cultured in the f/2 medium in filtered seawater (FSW) at room temperature under a photosynthetically active radiation (PAR) of 40 μmol m^−2^s^−1^ in a 12-h light/12-h dark (12L/12D) cycle. For treatment, three batch cultures were grown in the f/2 medium with temperatures 15 °C, 25 °C and 30 °C, respectively.

### Cell density determination

The *Symbiodinium* cell density was examined with haemocytometer based cell counting. Cell densities were determined daily by placing an aliquot of well-mixed culture suspension on a Neubauer hemocytometer (Marienfel, Germany) under an Axioskop2 Plus microscope (Zeiss, Germany) connected to a CCD camera (Photometrics, USA).

### The transmission electron microscopy and imaging analysis

To investigate the morphological variability of *Symbiodinium* cells within the host cells or in the free-living form, *Symbiodinium* cells were collected and fixed in 2.5% glutaraldehyde and 2% paraformaldehyde in 100 mM sodium phosphate containing 5% sucrose (pH 7.3) for 2.5 h at 4 °C. They were then rinsed with 100 mM sodium phosphate buffer at 4 °C. Cells were post-fixed in 1% OsO_4_ in 50 mM sodium phosphate (pH 7.3) for 1 h at 4 °C. The cell aliquots were then washed 3 times for 15 min each with the same buffer and dehydrated by a graded ethanol series (50, 70, 80, 90, 95 and 100%) before embedding in LR white Resin. Thin sections (70 nm) cut by a Leica Reichert Ultracut R were collected on nickel grids, post-stained with 2.5% uranyl acetate and 0.4% lead citrate, rinsed 3 times with water, and the samples were viewed on a JEM-1400 transmission electron microscope (JEOL, Japan). In order to determine the lipid droplet area from the acquired images, the ratio of the actual length to pixel was first determined by distance calibration using the scale bar of the acquired TEM image. Individual lipid droplet was selected by threshold adjustment, and the area (μm2) of each lipid droplet was calculated by using the region measurement function of Metamorph.

### Total protein extraction

The protocol was operated according to the work by Hurkman and Tanaka^43^. *Symbiodinium* cell was ground into powder in the mortar with liquid N_2_ The powder was mixed completely with 10 ml of the extraction buffer (0.7 M sucrose, 0.5 M Tris base, 30 mM HCl, 50 mM EDTA, 0.1 M KCl, and 2% β-mercaptoethanol) and 10 ml of phenol.  The upper phenol layer was separated from the lower buffer layer by centrifugation at 8,000 × g for 15 min at 4 °C and transferred the phenol layer into a new tube. To remove contaminants, the phenol layer was mixed with equal volume of fresh extraction buffer again. After complete mixing, the phenol layer was separated from the buffer again. After complete mixing, the phenol layer was separated from the buffer by centrifugation at 8,000 × g for 15 min at 4 °C and transferred into a new tube containing five-fold volume of pre-cold 0.1 M NH_4_OAc/methanol to precipitate the proteins at −20 °C overnight. The pellet was collected by centrifugation at 8,000 × g for 15 min at 4 °C and washed with 10 ml of 0.1 NH_4_OAc/methanol for three times followed by 10 ml of acetone for two times. Finally the pellet was dissolved in the lysis buffer (8M Urea, 2% CHAPS, 30 mm 1,4-dithiothretol (DTT)) and stored at −20 °C.

### Protein quantification

Proteins were quantified with the Quant-iT^TM^ protein assay kit (Invitrogen Molecular Probes, Italy), using the Qubit fluorometer according to the manufacturer’s instructions (the kit is used for quantify the protein concentrations ranging from 12.5 μg/ml to 5 mg/ml).

### SDS-PAGE

Protein extracted from the symbiotic *Symbiodinium cells* collected from *E. pulchella* and free-living *Symbiodinium* cells were suspended in an equal volume of 2 × sample buffer according to the suggestion in the Bio-Rad instruction manual and resolved by SDS-PAGE using 15% (w/v) polyacrylamide in the separating gel and 4.75% polyacrylamide in the stacking gel^44^. After electrophoresis, the gel was stained with Coomassie Blue R-250 and then destained.

### In-gel digestion of the major proteins in *Symbiodinium* cell

The several bands of *Symbiodinium* cell proteins resolved by SDS-PAGE were manually excised from the gel and ground into pieces. After washing with 50% acetonitrile and 50% acetonitrile/25 mM ammonium bicarbonate, the protein was reduced and alkylated at 56 °C for 45 min in 10 mM dithiothreitol and 55 mM iodoacetamide in 25 mM ammonium bicarbonate, followed by overnight in-gel digestion with 0.1 μg in 15 μl of TPCK-treated modified porcine trypsin (Promega, USA) in the same buffer at 37 °C. The supernatant containing tryptic peptides was combined with two more extracts of the gel by 50% acetonitrile/5% formic acid. The sample was analyzed by matrix-assisted laser desorption/ionization-mass spectrometry (MALDI-MS) and MALDI-MS/MS. All data were acquired by quadrupole-time-of-flight (Q-TOF) hybrid mass spectrometers (Micromass Q-Tof Ultima, Manchester, UK, and Applied Biosystems QSTAR, USA), in which α-cyano-4-hydroxycinnamic acid was used as the matrix. The low-energy collision-induced dissociation MS/MS product ion spectra acquired from Q-TOF Ultima and QSTAR were analyzed by Micromass ProteinLynx^TM^ Global Server 2.0 and Applied Biosystems BioAnalyst^TM^ data processing software, respectively. For protein identification, the acquired MS/MS spectra were automatically searched against the NCBInr database using the Mascot search program (www.matrixscience.com) restricted to all entries taxonomy. The mass tolerance parameter was 20 ppm, the MS/MS ion mass tolerance was 1 Da, and up to one missed cleavage was allowed. Variable modifications considered were methionine oxidation and cysteine carboxyamidomethylation. Positive identification of proteins was confirmed by observation of at lest one of the following criteria: (i) the total number of matched peptides (mps) is more than 2, or (ii) the mps equals 2 with two different matched peptides, or (iii) the MOWSE score has to be higher than 67 which indicates identity or extensive homology (p < 0.05).

## Additional Information

**How to cite this article**: Pasaribu, B. *et al.* Morphological Variability and Distinct Protein Profiles of Cultured and Endosymbiotic *Symbiodinium* cells Isolated from *Exaiptasia pulchella*. *Sci. Rep.* **5**, 15353; doi: 10.1038/srep15353 (2015).

## Supplementary Material

Supplementary Information

## Figures and Tables

**Table 1:** Different cell types between the freshly isolated Symbiotic cells and free-living cultured *Symbiodinium* cells.

	Symbiotic cells	Free living cultured cells
Cell size (diameter, μm)	6.49 ± 1.06^a^ (n = 688)	5.60 ± 0.02^b^ (n = 1392)
Cell wall (thickness, μm)	0.02 ± 0.0005^b^ (n = 470)	0.07 ± 0.001^a^ (n = 1048)
LDs number (per algal cell)	2.19 ± 1.54^a^ (n = 75)	0.80 ± 0.02^b^ (n = 605)

Impact of the nutrient regime in cell pattern between the symbiotic and free-living cultured *Symbiodinium* cells as analyzed by ANOVA. Upper-level letter, a and b, denote the different statistical significance between symbiotic cells and free-living cultured cells, respectively (P < 0.001). Cell wall, cell size: n = number of cell analyzed, LDs number: n = number of LDs analyzed.

**Table 2:** Functional classification of proteins identify from freshly isolated Symbiotic *Symbiodinium* cells.

Protein name	Species	BandNo.	GI NO.	PredictedMW(kDa)	ObservedMW(kDa)	score
*1. Transcription and Translation factors*						
⧫ EF-1 α-like protein	*Ostreococcus tauri*	I1	308820507	54.144	58	167
⧫ Probable WRKY transcription factor 30-like	*Brachypodium distachyon*	I2	357118663	36.852	32	75
⧫ DREB:Dehydration responsive element binding protein	Sorghum bicolor	I4	337263662	28.577	24	52
⧫ Pre-mRNA splicing factor ATP-dependent RNA helicase-like protein	Primula vulgaris	I5	82547933	18.367	20	51
*2. Photosystem and chlororphyll protein*						
⧫ PsbD: Photosystem II D2 protein	*Ceratophyllum demersum*	I1	11022876	36.095	58	85
⧫ Glutamate-1-semialdehyde aminotransferase	*Amaranthus tricolor*	I2	13676396	32.854	32	48
*3. Energy metabolism*						
⧫ NADH dehydrogenase subunit F	*Bassia prostrata*	I2	56694546	30.119	32	67
⧫ Oxidoreductase, short chain dehydrogenase/ reductase family protein	*Oryza sativa Japonica Group*	I2	108863020	31.081	32	54
⧫ ARF-related small GTPase	*Chlamydomonas reinhardtii*	I5	159466722	21.297	20	43
*4. Lipid metabolism*						
⧫ Acot13/acyl-coA thioesterase 13	*Brachypodium distachyon*	I3	357128623	17.735	24	57
⧫ 7-dehydrocholesterol reductase-like	*Oryza sativa Japonica Group*	I4	125539389	31.195	22	75
*5. Defense response*						
⧫ LRR-kinase protein	*Glycine max*	I5	47088292	13.557	20	45
