# Supplementary material for: Morphological Variability and Distinct Protein Profiles of Cultured and Endosymbiotic Symbiodinium cells Isolated from Exaiptasia pulchella
Source: Sci Rep. 2015 Oct 20;5:15353. doi: 10.1038/srep15353 (PMC4611179; doi:10.1038/srep15353)
Supplement: Supplementary Information [file srep15353-s1.pdf]

Morphological Variability and Distinct Protein Profiles of Cultured and Endosymbiotic *Symbiodinium* cells Isolated from *Exaiptasia pulchella*

Buntora Pasaribu[3§], Li-Chi Weng[1§], I-Ping Lin[6], Eddie Camargo [3], Jason T. C. Tzen[3], Ching-Hsiu Tsai[3], Shin-Lon Ho[5], Mong-Rong Lin[1], Li-Hsueh Wang [1,2] , Chii-Shiarng Chen[1,2,4*] & Pei-Luen Jiang [1,2]*

[1]Graduate Institute of Marine Biology, National Dong-Hwa University, Pingtung, 944 Taiwan

[2]Taiwan Coral Research Center, National Museum of Marine Biology and Aquarium, Pingtung 944, Taiwan

[3] Graduate Institute of Biotechnology, National Chung-Hsing University, Taichung 402, Taiwan

[4]Department of Marine Biotechnology and Resources, National Sun Yat-Sen University, Kaohsiung 804, Taiwan

[5]Department of Agronomy, National Chia-Yi University, Chia-Yi 600, Taiwan

[6]Department of Biotechnology, National Formosa University, Taiwan

* To whom correspondence should be addressed

*Corresponding author.

Chii-Shiarng Chen

Tel: +886-8-8825040; Fax: 886-8-8825087; E-mail: cchen@nmmba.gov.tw

Pei-Luen Jiang, Institute of Marine Biology, National Dong Hwa University and National Museum of Marine Biology and Aquarium, 2 Houwan Road, Checheng, Pingtung 944, Taiwan. Tel: 886-8-8825046; Fax: 886-8-8825087; E-mail: villy@nmmba.gov.tw

([§] These authors contributed equally to this work.)

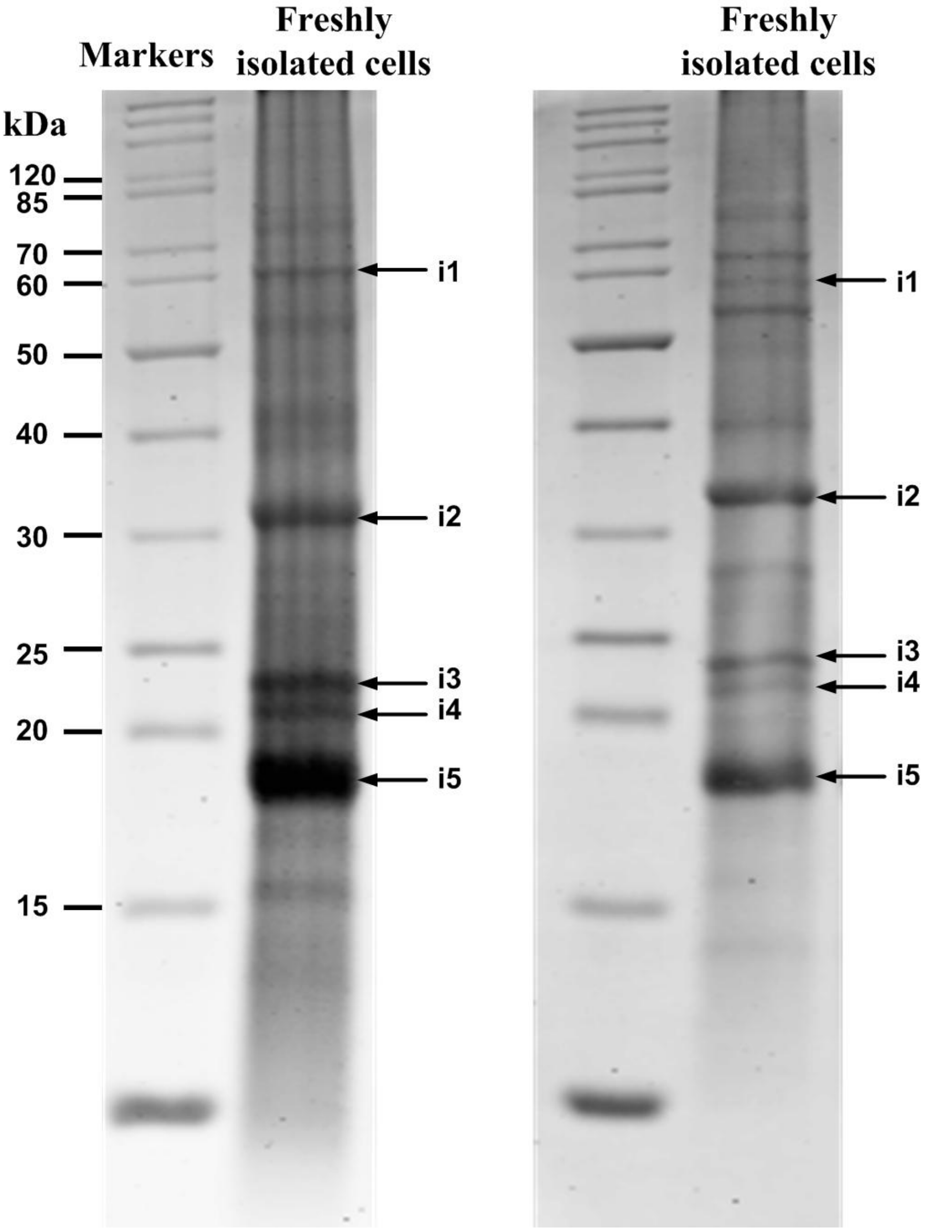

Supplementary data

Figure S1 **Protein expressed in freshly isolated symbiotic *Symbiodinium* cells.** Total protein was extracted from freshly isolated *Symbiodinium* sp. resolved in SDS-PAGE.
